# Remote Administration of ADHD-Sensitive Cognitive Tasks: A Pilot Study

**DOI:** 10.1177/10870547231172763

**Published:** 2023-06-02

**Authors:** Shaoxiong Sun, Hayley Denyer, Heet Sankesara, Qigang Deng, Yatharth Ranjan, Pauline Conde, Zulqarnain Rashid, Rebecca Bendayan, Philip Asherson, Andrea Bilbow, Madeleine Groom, Chris Hollis, Amos A. Folarin, Richard J. B. Dobson, Jonna Kuntsi

**Affiliations:** 1 King’s College London, UK; 2 ADDISS, The National Attention Deficit Disorder Information and Support Service, Edgware, Middlesex, UK; 3 University of Nottingham, UK; 4 University College London, UK; 5 NIHR Biomedical Research Centre at University College London Hospitals NHS Foundation Trust, UK

**Keywords:** ADHD, remote monitoring, attention regulation, response inhibition, RADAR-base

## Abstract

**Objective:**

We assessed the feasibility and validity of remote researcher-led administration and self-administration of modified versions of two cognitive tasks sensitive to ADHD, a four-choice reaction time task (Fast task) and a combined Continuous Performance Test/Go No-Go task (CPT/GNG), through a new remote measurement technology system.

**Method:**

We compared the cognitive performance measures (mean and variability of reaction times (MRT, RTV), omission errors (OE) and commission errors (CE)) at a remote baseline researcher-led administration and three remote self-administration sessions between participants with and without ADHD (*n* = 40).

**Results:**

The most consistent group differences were found for RTV, MRT and CE at the baseline researcher-led administration and the first self-administration, with 8 of the 10 comparisons statistically significant and all comparisons indicating medium to large effect sizes.

**Conclusion:**

Remote administration of cognitive tasks successfully captured the difficulties with response inhibition and regulation of attention, supporting the feasibility and validity of remote assessments.

## Introduction

Extensive past research links attention deficit hyperactivity disorder (ADHD), in both children and adults, to a cognitive profile that indicates difficulties with the regulation of attention and aspects of executive functioning, such as response inhibition (Franke et al., 2018; Pievsky & McGrath, 2018). Cognitive tasks that are particularly sensitive to differences between people with and without ADHD include the Continuous Performance Test (CPT) and the Go/No-Go (GNG) task, as well as simple four-choice reaction time tasks (Cheung et al., 2016; Kuntsi et al., 2010).

While cognitive task administration typically involves the participant attending an assessment session at a clinic or a research center, the possibility of remote administration of cognitive tasks has immense appeal. Remote self-administration of cognitive tasks would enable long-term monitoring for either research or clinical purposes. The Covid-19 pandemic has further illustrated the importance of remote data collection methods for times when in-person visits to clinics may not be feasible. Diversity and inclusion of participants can also improve, as remote assessments are more accessible to less mobile individuals or those living in isolated areas. Yet remote self-administration of cognitive tasks is not without challenges; its validity must be demonstrated.

We have recently developed a new remote measurement technology (RMT) system for adults and adolescents (ages 16+) with ADHD that also incorporates two cognitive tasks for remote self-administration. The initial goal for the development of the ADHD Remote Technology (ART) system is to enable long-term, real-world monitoring of symptoms, impairments, and health-related behaviors. ART is linked to the open source mobile-health platform RADAR-base (Ranjan et al., 2019; Stewart et al., 2018) and consists of both active and passive monitoring using mobile and web technologies. Passive monitoring, which requires no active input from the participant, is continuous using smartphone sensors and a wearable device. Active monitoring involves the participant completing questionnaires or other tasks on a smartphone Active App, and the self-administration of two cognitive tasks using a laptop or PC.

The cognitive tasks in the ART system consist of modified versions of the Fast task and a combined CPT/GNG task. The Fast task is a four-choice reaction time task that probes attention regulation (see Methods for a full description of the task). Individuals with ADHD show increased reaction time variability (RTV) on the task, which is associated with neurophysiological measures of attention allocation and arousal dysregulation (Cheung et al., 2017; James et al., 2016). Mean reaction time (MRT) on the task is also slower among individuals with ADHD. Our previous research indicates very high phenotypic and genetic/familial correlations (.8–.9) between RTV and MRT, suggesting they capture largely the same process on both the Fast task and the GNG task (Kuntsi et al., 2010, 2014). The combined CPT/GNG task probes attentional processes (omission errors (OE), RTV) and response inhibition (commission errors (CE)). The version we have developed for ART is a modified version based on a “CPT-OX” task we have used previously in studies on ADHD (Cheung et al., 2016; McLoughlin et al., 2010). It consists of a low target probability condition (“CPT”: 1:5) and a high target probability condition (“GNG”: 5:1). Our previous larger-scale studies have indicated differences between ADHD and comparison groups on OE, CE, MRT, and RTV on CPT-OX and GNG tasks (Cheung et al., 2016; Kuntsi et al., 2010), while in our smaller-scale study using the CPT-OX group differences emerged for OE, MRT and RTV, but not for CE (although a medium effect size was observed for the latter too; [McLoughlin et al., 2010]). Overall, the Fast and CPT/GNG tasks enable measurement of cognitive performance differences in people with ADHD that are linked to genetic risk for ADHD (Kuntsi et al., 2010; Vainieri et al., 2021) and capture markers of ADHD persistence/remission (James et al., 2016, 2020; Michelini et al., 2016, 2019; Vainieri et al., 2020).

As part of a 10 week remote monitoring pilot study on the ADHD Remote Technology (ART) system that involved an initial baseline remote administration by researcher and three subsequent remote self-administrations of the cognitive tasks, we addressed the following research questions and tested the following hypotheses:

1. *Remote administration by researcher*: Does remote administration of the Fast task and the CPT/GNG task by a researcher produce the ADHD-control differences expected based on past research? Our hypothesis is that participants with ADHD perform less well than control participants on each of the outcome variables (RTV, MRT, CE, and OE) at baseline administration by the researcher. The pilot study was carried out during the Covid-19 pandemic, which required remote (rather than in-person) baseline assessments by the researcher. The demonstration of the validity of such remote assessments is therefore the first step.
2. *Remote self-administration*: Does remote self-administration of the Fast task and the CPT/GNG task produce similar ADHD-control differences as observed during the baseline researcher-administration of the tasks? Our hypothesis is that participants with ADHD perform less well than control participants on each of the outcome variables (RTV, MRT, CE, and OE) at study week 2 (first self-administration).
3. *Group differences at study weeks 6 and 10*: Are similar ADHD-control group differences still observed at study weeks 6 and 10? We have no directional hypothesis here, as it is uncertain how participants may cope with the repeated administration of these typical ADHD tasks that are designed to be challenging for people with ADHD and are therefore purposefully rather monotonous.

## Methods

### Participants

We recruited 20 individuals with ADHD and 20 control participants between the ages of 16 and 39 into the study (Table 1). Participants were recruited from previous studies (where they had indicated that they were willing to be contacted regarding future research studies), via the Attention Deficit Disorder Information and Support Service (ADDISS), social media, King’s Volunteer circular and on the “Call for Participants” website (https://www.callforparticipants.com/). Exclusion criteria for the individuals with ADHD were: (1) having psychosis, major depression, mania, drug dependency, or a major neurological disorder, (2) any other major medical condition which might impact upon the individual’s ability to participate in normal daily activity, (3) pregnancy, (4) IQ of <70 and (5) not currently taking medication for their ADHD. Exclusion criteria for the control group were: (1) meeting diagnostic criteria for ADHD based on the self-report on Barkley Adult ADHD Rating scale on current symptoms (BAARS-IV) and Barkley ADHD functional impairment questionnaire, (2) having psychosis, major depression, mania, drug dependency, or a major neurological disorder, (3) any other major medical condition which might impact upon the individual’s ability to participate in normal daily activity, (4) pregnancy, and (5) IQ of <70.

**Table 1.** Demographics Divided by Group, with Tests for Differences Between Participants With ADHD and Controls.

	ADHD (*n* = 20)	Control (*n* = 20)	*p*-Value
Gender, female %	75	75	1.0
Age, mean (*SD*)	27.49 (6.04)	27.79 (6.17)	.88
WASI-II vocabulary subscale, mean (*SD*)	57.85 (7.53)	56.80 (8.35)	.68

The study was approved by the North East—Tyne and Wear South Research Ethics Committee (REC reference: 20/NE/0034). Informed consent was obtained from participants before the assessments started. Participants were compensated £30 after completion of the baseline sessions, £20 after the first remote active monitoring follow-up (end of week 5) and a further £50 at study endpoint (end of week 10).

### Procedure

ART-pilot is an observational non-randomized, non-interventional study, using commercially available wearable technology and smartphone sensors, representing no change to the usual care or treatments of participants due to participation.

Participants attended two remote baseline sessions with a research worker, using Microsoft Teams. The first remote baseline session with the participants with ADHD included the administration of the following assessments: (1) the Diagnostic Interview for ADHD (DIVA) in adults (Kooij & Francken, 2010) to confirm ADHD diagnosis, (2) vocabulary and digit span subscales from the Wechsler Abbreviated Scale of Intelligence (WASI-II) (Wechsler, 1999) and Wechsler Adult Intelligence Scale (WAIS-IV; Wechsler et al., 2008), respectively, and (3) web-based REDCap (https://projectredcap.org) baseline questionnaires. The second session was administered once participants had received their wearable device and smartphone by post, approximately a week after the first session. The second session included: (1) administration of two cognitive tasks (the Fast task and the combined cued continuous performance test (CPT-OX) and Go/NoGo (GNG) task), and (2) a training session on the use of the wearable device, and a smartphone Passive and Active App. The participant also received a leaflet summarizing key information (Participant Technology User Guide) and researcher contact details for future reference. Each session lasted for approx. 1.5 hr. Control participants were assessed in the same way, except that instead of the full ADHD diagnostic interview, they completed the ADHD symptom and impairment questionnaire (Barkley & Murphy, 2006).

Immediately following the second baseline session, participants then took part in a 10-week remote monitoring period, which included passive and active monitoring measures. Passive monitoring involved using the RADAR-base smartphone Passive App and a wrist-worn activity monitor (Fitbit Charge 3) (Sun et al., 2020, 2022; Zhang et al., 2021, 2022).

Active monitoring involved the participant completing clinical symptom questionnaires on the RADAR-base smartphone Active App, which is beyond the scope of this current analysis, and the two cognitive tasks on their home PC or laptop, and took place three times: at 2 weeks (the first remote self-administrated assessment), 6 weeks (the second remote self-administrated assessment) and 10 weeks (the third remote self-administrated assessment) after the baseline remote researcher-led session. We asked participants to complete the cognitive tasks in a quiet environment, free from distraction. Participants received an event notification on the Active App, which reminded the participant that it was time to complete their questionnaires and the cognitive tasks. Participants were asked to complete the cognitive tasks within 3 days of receiving the event notification. However, where the participant had to delay completing the tasks (e.g., until the weekend), the data were included in the analysis. Each participant was in the study for 10 weeks.

### Measures

#### The Fast Task

The Fast task is a computerized four-choice RT task, which measures performance under a slow-unrewarded and a fast-incentive condition (Andreou et al., 2007; Kuntsi et al., 2006). In both conditions speed and accuracy were emphasized equally. The baseline (slow unrewarded) condition followed a standard warned four-choice reaction-time task. A warning signal (four empty circles, arranged side by side) first appeared on the screen. At the end of the fore-period lasting 8 s (presentation interval for the warning signal), the circle designated as the target signal for that trial was filled (colored) in. The participant was asked to make a compatible choice by pressing the response keys (F, G, H, and I using a QWERTY keyboard) that directly corresponded in position to the location of the target stimulus. Following a response, the stimuli disappeared from the screen and a fixed inter-trial interval of 2.5 s followed. If the participant did not respond within 10 s, the trial terminated. First, a practice session was administered, during which the participant had to respond correctly to five consecutive trials. For the ART program we shortened the baseline condition from 72 trials (used in our previous research) to 58 trials, equating to approx. 5 min reduction in length of administration. The task further includes a second condition that uses a fast event rate (fore-period of 1 s) and incentives. This condition started immediately after the baseline condition and consisted of 80 trials, with a fixed inter-trial interval of 2.5 s following the response. The baseline condition and the fast-incentive condition of the Fast task took approximately 5 and 10 min to complete, respectively. The participants were told to respond as quickly as possible to each target. Correct responses within a specified time window were followed by a feedback stimulus, a smiley face, indicating a correct response. Participants were rewarded with this smiley face feedback for responding faster than their own MRT during the baseline (first) condition consecutively for three trials. The smiley faces appeared below the circles in the middle of the screen and were updated continuously. We obtained performance measures of mean reaction time (MRT) and reaction time variability (RTV; SD of RTs). Performance measures were calculated on correctly answered trials only.

#### Combined Cued Continuous Performance Test (CPT-OX) and Go/NoGo (GNG) Task

The combined CPT/GNG task probes attention and response inhibition. The version we have developed for ART is partly based on a CPT-OX task we have used previously (Cheung et al., 2016; Doehnert et al., 2008; Valko et al., 2009) but further incorporates two conditions that differ by the target to non-target ratio: a low target probability condition (“CPT”: 1:5) and a high target probability condition (“GNG”: 5:1). The test consists of 400 letters presented for 150 ms with a stimulus onset asynchrony of 1.65 s in a pseudo-randomized order. The CPT and GNG conditions took approximately 11 min each to complete. Participants were instructed to press a space bar with the index finger of their dominant hand as fast as possible every time the cue was followed directly by the letter X [(O–X) target sequence] but had to withhold responses to O-not-X sequences (NoGo trials). Speed and accuracy were emphasized equally. We obtained performance measures of MRT, RTV, commission errors (CE) and omission errors (OE). MRT and RTV were calculated across correctly answered Go trials; CE were responses to Cue, NoGo and distractor stimuli or Go stimuli not following a Cue; and OE were non-responses to Go trials.

#### Barkley Adult ADHD Rating Scale on Current Symptoms (BAARS-IV) and Barkley ADHD Functional Impairment Scale (BFIS)—Self-Report Form

The BAARS-IV is an empirically developed self-report measure, based on DSM diagnostic criteria, for assessing current ADHD symptoms (Barkley, 2011a; Barkley & Murphy 2006). The scale includes the 18 diagnostic ADHD symptoms (nine items in each domain of inattention and hyperactivity/impulsivity), with a reported alpha of .92. The responses for each item are scored on a 4-point rating scale (0 = “never or rarely,” 1 = “sometimes,” 2 = “often,” and 3 = “very often”). The 18 items in the scale are arranged so that symptoms associated with inattention are the odd-numbered items and the hyperactive-impulsive symptoms are even-numbered. Inattention symptoms and hyperactive-impulsive symptoms should be scored separately. Symptoms are recorded as present if answered as “often” (2) or “very often” (3). For the present study, and consistent with DSM-V criteria, a symptom count of five or more items for inattention or hyperactivity-impulsivity is required.

The BFIS is a 10-item scale used to assess the level of functional impairments commonly associated with ADHD symptoms in five areas of everyday life: family/relationship, work/education, social interaction, leisure activities, and management of daily responsibilities. The BFIS has a reported alpha of .92 (Barkley, 2011b). The responses for each item are scored on a 4-point rating scale (0 = “never or rarely,” 1 = “sometimes,” 2 = “often,” and 3 = “very often”). To calculate the BFIS self-report total score, similar to the BAARS-IV, functional impairment is recorded as present if answered as “often” (2) or “very often” (3). For the BAARS-IV to be suitable as a monitoring measure, we changed the wording from “during the past 6 months” to “during the past 2 weeks” for each item.

#### The Diagnostic Interview for ADHD in Adults (DIVA)

The Diagnostic Interview for ADHD in Adults (DIVA) is a validated structured interview for ADHD (Kooij & Francken, 2010). The DIVA was conducted by trained researchers to assess DSM-5 criteria for adult ADHD symptoms and impairment. The DIVA is divided into categories of inattention symptoms, hyperactive-inattention symptoms, and impairments. For each of these areas, questions are asked about current symptoms and symptoms experienced in childhood (ages 5–12). Each item is scored affirmatively if the behavioral symptom was present “often” within the past 6 months.

#### Verbal IQ

The vocabulary subtest of the Wechsler Abbreviated Scale of Intelligence (WASI-II) (Wechsler, 2011) was administered to all participants to derive an estimate of verbal IQ.

#### Short-Term and Working Memory Assessment

The digit span subtest of the Wechsler Adult Intelligence Scale (WAIS-IV; Wechsler et al., 2008) was administered to all participants to measure short-term verbal memory (digit span forward) and working memory (digit span backward).

### Statistical analyses

We used independent *t*-tests to examine group differences in age and WASI-II vocabulary subscale, and the chi-square test to test for a group difference in gender. We compared the performance measures in the cognitive tasks at four time points (baseline, week 2, week 6, and week 10) between participants with ADHD and controls. Specifically, we calculated MRT, RTV, OE, and CE for the combined conditions of the CPT/GNG task. Similarly, we calculated MRT and RTV for the combined conditions of the Fast task. The exclusion criteria were uncompleted tasks, implausible reaction time (<150 ms), and proportion of correct responses lower than 50% (for the CPT/GNG task). The number of excluded data points is given in Table 2. Normality of the derived performance measures was examined using the Shapiro-Wilk test for its power in detecting non-normality, where *p*-value ≥ .05 was considered normal distribution (Wah & Razali, 2011). Given that the data in the two groups at four time points were not all normally distributed, the Wilcoxon rank-sum tests were used to ensure robustness with our sample sizes and distributional characteristics (non-normal data). A statistically significant difference was defined as *p*-value < .05. In order to inform future larger-scale studies, multiple testing corrections were not undertaken to reduce the chance of type-two errors (i.e., false negative results) and to avoid over correction for multiple comparisons involving multiple correlated variables. Effect sizes were estimated as the standardized *Z* value divided by the total number of samples for the Wilcoxon rank-sum test. For small, medium and large effect size, the required thresholds are 0.1, 0.3, and 0.5, respectively (Fritz et al., 2012). To quantify the variability of these measures over time for each feature, the coefficient of variation was calculated as the standard deviation of four time points of the group-medians divided by its respective mean of the group-medians. All the statistical analyses were implemented in Python 3.7.4.

**Table 2.** Data Exclusion Grouped by Tasks and Exclusion Criteria.

Task	Criteria	ADHD	Control
Fast task	Uncompleted task	14 (17.5%)^a^	1 (1.3%)
	Implausible reaction time^b^	0	1 (1.3%)
CPT/GNG	Uncompleted task	16 (20%)	3 (3.9%)
	Implausible reaction time	0	1 (1.3%)
	Low proportion of correct responses^c^	3 (3.9%)	0

a Out of the 80 tasks put together at four time points completed by 20 participants

b Implausible reaction time (<150 ms)

c Proportion of correct responses lower than 50%.

## Results

The groups did not differ in gender, age, or verbal IQ (Table 1).

**Figure 1.**
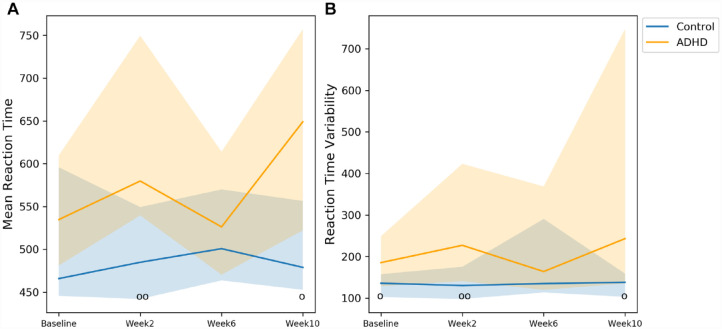
Comparisons between participants with ADHD and controls in the Fast task. The markers o and oo represent *p* < .05 and *p* < .01, respectively. Solid line: median; shade: 25th percentile to 75th percentile.

On the Fast task, significant group differences were detected in RTV at baseline, in MRT and RTV at week 2, and in MRT and RTV at week 10 (Figure 1 and Table 3). The corresponding effect sizes were all medium. The coefficient of variation for MRT were 0.09 and 0.03 and for RTV 0.15 and 0.02, for participants with ADHD and controls, respectively.

**Table 3.** *p*-Values, Effect Sizes and Numbers of Participants in each Comparison in the Fast Task.

Variable	*p*-Value	Effect size	ADHD/Controls^a^	Median (IQR)^b^
Baseline				
MRT^c^	.10	.26	20/19	534.76 (128.62)/465.91 (150.12)
RTV^d^	**.02** *	.37	20/19	185.44 (120.35)/135.92 (55.03)
Week 2				
MRT	**.004** *	.47	17/20	579.83 (209.84)/484.87 (107.28)
RTV	**.006** *	.44	17/20	227.14 (282.25)/130.44 (77.38)
Week 6				
MRT	.55	.10	14/20	526.25 (143.55)/500.8 (106.24)
RTV	.48	.12	14/20	163.93 (248.83)/135.07 (176.86)
Week 10				
MRT	**.03** *	.37	15/19	649.11 (234.93)/478.92 (103.72)
RTV	**.03** *	.38	15/19	243.18 (611.90)/138.16 (55.75)

a Number of participants who completed the tasks.

b Median and interquartile range (IQR) of participants with ADHD and controls.

c Mean reaction time.

d Reaction time variability.

* Statistically significant p<0.05

**Figure 2.**
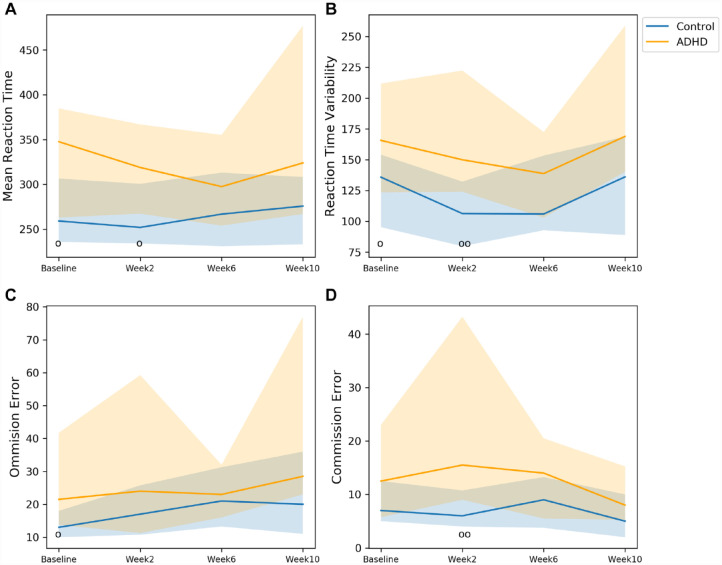
Comparisons between participants with ADHD and controls in the CPT/GNG task. The markers o and oo represent *p* < .05 and *p* < .01, respectively. Solid line: median; shade: 25th percentile to 75th.

On the CPT/GNG task, significant group differences were found at baseline for MRT, RTV, and OE, at week 2 for MRT, RTV, and CE. (Figure 2 and Table 4). Effect sizes for these significant group differences ranged from medium to large. Although not statistically significant, the group differences in CE at baseline and RTV at week 10 exhibited medium effect sizes. The coefficient of variation for MRT were 0.06 and 0.03, for RTV 0.08 and 0.12, for OE 0.11 and 0.18 and for CE 0.22 and 0.22 for participants with ADHD and controls, respectively.

**Table 4.** *p*-Values, Effect Sizes and Numbers of Participants in the CPT/GNG Task.

Variable	*p*-Value	Effect size	ADHD/Controls^a^	Median (IQR)^b^
Baseline				
MRT^c^	**.02** *	.37	18/19	347.68 (121.88)/259.12 (70.84)
RTV^d^	**.02** *	.39	18/19	165.73 (88.41)/135.86 (58.86)
OE	**.02** *	.39	18/19	21.5 (28.0)/13.0 (8.0)
CE	.07	.30	18/19	12.5 (17.25)/7.0 (7.5)
Week 2				
MRT	**.02** *	0.39	16/20	318.92 (99.57)/251.97 (66.50)
RTV	**.007** *	.45	16/20	150.02 (98.39)/106.3 (52.41)
OE	.26	0.19	16/20	24.0 (48.0)/17.0 (15.0)
CE	**.002** *	.51	16/20	15.5 (34.25)/6.0 (6.75)
Week 6				
MRT	.29	.18	15/20	297.53 (101.28) /266.80 (82.11)
RTV	.19	.22	15/20	138.82 (69.50)/105.98 (60.67)
OE	.50	.11	15/20	23.0 (16.0)/21.0 (18.0)
CE	.13	.25	15/20	14.0 (15.0)/9.0 (9.5)
Week 10				
MRT	.11	.30	12/17	323.99 (210.21)/275.78 (75.19)
RTV	.06	.35	12/17	168.98 (118.57)/136.12 (79.88)
OE	.14	.28	12/17	28.5 (54.0)/20.0 (25.0)
CE	.29	.20	12/17	8.0 (10.0)/5.0 (8.0)

a Number of participants who completed the tasks.

b Median and interquartile range (IQR) of participants with ADHD and controls.

c Mean reaction time.

d Reaction time variability.

* Statistically significant p<0.05

## Discussion

As part of a pilot study using our new ADHD Remote Technology (ART) system, we show that both remote researcher-led administration and self-administration of cognitive tasks capture the difficulties people with ADHD have with the regulation of attention and response inhibition, supporting the feasibility and validity of remote assessments. Reaction time variability (RTV), mean reaction time (MRT) and commission errors (CE) were the variables most consistently sensitive to ADHD-control group differences at the remote baseline researcher-led administration and the first remote self-administration, with medium to large effect sizes (statistically significant for 8 of the 10 comparisons). Subsequent remote self-administrations of the tasks showed that group differences became small and non-significant at week 6, but emerged again as significant for the Fast task RT variables at week 10.

The remote researcher-led cognitive assessments indicated the expected ADHD-control group differences (Cheung et al., 2016; Kuntsi et al., 2010; McLoughlin et al., 2010) on nearly all cognitive variables at baseline. On the CPT/GNG task, participants with ADHD performed

less well than control participants on RTV, MRT, and OE, with the CE result further at trend-level (*p* = .07); all effect sizes were medium. On the Fast task, a significant group difference, with a medium effect size, emerged for RTV. Overall, these results indicate that remote researcher-led task administration of the Fast task and CPT/GNG task is a valid alternative to the traditional in-person task administration with adults and young people with and without ADHD.

The first remote self-administration (week 2) produced overall similar ADHD-control differences as observed for the researcher-led baseline administration. At week 2, the group comparisons on the CPT/GNG task RTV and MRT, and Fast task RTV remained significant; the OE result was no longer significant, however. In addition, the group comparisons on the Fast task MRT and CPT/GNG task CE were now significant too. Effect sizes were medium to large. This illustration of how remote self-administration of cognitive tasks can successfully capture the difficulties with attention regulation and response inhibition in adults and young people with ADHD is highly promising for future remote measurement studies. Aspects of the set up and research process that may have contributed toward this successful outcome include: the training on task administration provided as part of the baseline researcher-led session, and clear written task instructions.

Further repeated remote self-administrations of the tasks resulted in significant group differences at week 10, but not at week 6. For the Fast task, group comparisons for both RTV and MRT were significant at week 10, with medium effect sizes. For the CPT/GNG task, no group comparison was statistically significant at week 10, but medium effect sizes emerged also for MRT and RTV (with *p*-values of .11 and .06). Due to the lower numbers of completed tasks at week 10, with data available from only 12 participants with ADHD, the statistical power for the week 10 CPT/GNG task analyses would be substantially reduced for MRT and RTV. The overall variability in these results of showing group differences only at week 10 and not at week 6 might relate to factors that influence how well participants cope with repeated administration of tasks that are purposefully rather monotonous. Future research with larger sample sizes may investigate this variability further.

A limitation of the study is the modest sample sizes and that some of the remote monitoring data were missing, particularly for the ADHD group. The sample sizes were adequate for this pilot study, as illustrated by the many significant group differences despite the modest statistical power, reflecting the medium to large effect sizes. Future studies with larger sample sizes are required to establish whether significant group differences emerge with improved statistical power for those variables (especially CE) that did not pick up significant group differences in this pilot study. Another limitation of the study is that we were unable to randomize the order with which the cognitive tasks were presented due to our programing settings. We have improved this in our new, ongoing European Commission-funded clinical study “ART-CARMA” (ADHD Remote Technology study of cardiometabolic risk factors and medication adherence; Denyer et al., 2022b), which involves remote monitoring of 300 adults with ADHD over a period of 12 months.

The reasons why some of the remote monitoring data were missing particularly for the ADHD group are of interest. We completed endpoint debrief interviews with participants who took part in the pilot study to understand the barriers and facilitators to RMT (Denyer et al., 2022a, Denyer et al., in press). The interviews have been analyzed using thematic analysis, and barriers and facilitators to RMT have been compared between individuals with ADHD and control participants. More individuals with ADHD described the cognitive tasks as a “perceived cost” compared to the control group. This finding would be expected due to the demand of the cognitive tasks on individuals who have difficulties with attention on demanding tasks. That is, the cognitive tasks have been specifically designed to be challenging for individuals with ADHD. Overall, therefore, an implication for future remote monitoring studies is to consider longer intervals in between repeated administrations of relatively long cognitive tasks, such as the ones employed here. We have already incorporated this consideration in the “ART-CARMA” study where the remote self-administration of the cognitive tasks takes place at 6-month intervals (Denyer et al., 2022b).

Another example of a topic that can particularly benefit, in the future, from remote long-term monitoring of people with ADHD that includes cognitive tasks is the investigation of cognitive differences that are markers of remission (improve when ADHD remits) versus those that are enduring differences (observed in individuals with a past ADHD diagnosis irrespective of later outcome). Using the Fast task and a CPT-OX task, our previous ADHD follow-up study indicated that attention-vigilance measures, including RTV, were markers of remission, whereas executive control measures were not sensitive to ADHD outcomes (persistence/remission; (James et al., 2016, 2020; Michelini et al., 2016, 2019; Vainieri et al., 2020). IQ further moderated ADHD outcome. Yet the data from our study and most other previous follow-up studies (Franke et al., 2018) are limited to few (mostly just two) time points: we know little about whether any changes in cognitive impairments are stable or temporary over time. As we now show feasibility and validity for home self-administration of the cognitive tasks, this method enables future remote administration in future studies on persistence and remission of ADHD.

Remote monitoring technology and the mobile-health platform infrastructures supporting them are bringing major advances and opportunities for longitudinal research. One advantage is the possibility to collect data simultaneously, in the real world, on a wide range of both novel and conventional measures. Our data here show that remote self-administration of cognitive tasks, designed for ADHD research, is a feasible part of such remote data collection for studies on adults and young people with ADHD, supporting their inclusion in the ART system.
